# Third party, umbilical cord blood derived regulatory T-cells for prevention of graft versus host disease in allogeneic hematopoietic stem cell transplantation: feasibility, safety and immune reconstitution

**DOI:** 10.18632/oncotarget.26242

**Published:** 2018-11-02

**Authors:** Joshua N. Kellner, Eveline M. Delemarre, Eric Yvon, Stefan Nierkens, Jaap J. Boelens, Ian McNiece, Amanda Olson, Yago Nieto, Stefan Ciurea, Uday Popat, Sairah Ahmed, Richard Champlin, Jennifer Ramos, Mitsutaka Nishimoto, Hongbing Ma, Zeng Ke, Peter Thall, Joseph D. Khoury, Robert Negrin, Borje Andersson, Simrit Parmar

**Affiliations:** ^1^ Department of Experimental Therapeutics, University of Texas MD Anderson Cancer Center, Houston, TX, USA; ^2^ Laboratory of Translational Immunology, University Medical Center Utrecht, Heidelberglaan, CX Utrecht, The Netherlands; ^3^ Department of Stem Cell Transplantation and Cellular Therapy, University of Texas MD Anderson Cancer Center, Houston, TX, USA; ^4^ Department of Pediatrics, Stem Cell Transplant and Cellular Therapies, Memorial Sloan Kettering Cancer Center, New York, NY, USA; ^5^ Department of Lymphoma and Myeloma, University of Texas MD Anderson Cancer Center, Houston, TX, USA; ^6^ Department of Biostatistics, University of Texas MD Anderson Cancer Center, Houston, TX, USA; ^7^ Department of Pathology, University of Texas MD Anderson Cancer Center, Houston, TX, USA; ^8^ Division of Blood and Marrow Transplantation, Stanford University, Palo Alto, CA, USA

**Keywords:** regulatory T cells, umbilical cord blood, graft versus host disease, cell therapy, stem cell transplantation

## Abstract

Incubation of umbilical cord blood (UCB) derived regulatory T-cells (Tregs) with fucosyltransferase enzyme improves their ability to home to the target tissue to prevent graft vs. host disease (GVHD). We report results of 5 patients (Double UCB Transplant, n=2; Peripheral Blood Matched Unrelated Donor Transplant, n=3) who received UCB-Tregs (Dose level = 1×106/kg), infused one day prior to the donor graft. All patients received their designated UCB-Treg dose without any infusion reaction. The ratio of conventional T-cells in donor graft was at least 10 times higher than infused UCB-Tregs (ratio range, 12-356). All patients engrafted at median of 13 days (range, 8-17 days). One patient died due to brain hemorrhage on day 45. A bi-modal increase of plasma IL-10 level occurred on day 7 and day 21 and notably, plasma IL-2 level dropped significantly in all patients at Day 7. All evaluable patients developed ≥grade II acute GVHD and at 1 year follow up, all were alive and without evidence of disease relapse. No increase in the chronic GVHD biomarkers (REG3a and Elafin) was observed at day 7. At the time of last follow up, all evaluable patients were off immune-suppression. Stage 2 of this clinical trial examining UCB-Treg at dose level= 1×107/kg is currently underway.

## INTRODUCTION

We previously showed that 3^rd^ party, *ex-vivo* expanded, umbilical cord blood (UCB) Treg cells can prevent graft versus host disease (GVHD) in xenogenic mouse model [1]. Additionally, efficacy of cultured UCB Tregs improves when incubated with fucosyltransferase-VI enzyme, which establishes Siayl-Lewis X moiety on P-selectin [2]. We hypothesized that adoptive therapy with fucosylated UCB Tregs would prevent GVHD and conducted a pilot clinical trial (https://www.clinicaltrials.gov NCT02423915). We report preliminary safety data in 5 patients undergoing allogeneic stem cell transplant (AlloSCT) (Double UCB Transplant (dUCBT)= 2; Peripheral Blood (PB) Matched Unrelated Donor Transplant (MUD) = 3) who received UCB Tregs at dose: 1×10^6^ cells/kg (Fucosylated UCB Tregs = 3; Non-Fucosylated UCB Tregs = 2) that were matched at least at HLA 3/6 to recipient.

## RESULTS

### Graft and UCB Treg characteristics

Five patients were treated at UCB Treg dose level: 1×10^6^ cells/kg; 2 patients received non-fucosylated UCB Tregs followed by dUCB AlloSCT and 3 patients received fucosylated UCB Tregs followed by PB MUD AlloSCT. Donor graft and UCB Treg characteristics are shown in Table 1. All patients received designated UCB Treg dose: 1×10^6^ cells/kg (1.16×10^6^/kg ±0.05) and purity of UCB Treg product (phenotype:CD4^+^25^+^127^lo^) at the time of release and infusion on day 14 of expansion was 90% (range, 86-93%). UCB units identified for Treg manufacture had median of 9.6×10^8^ TNCs (range, 9.1-11.4×10^8^ TNCs) with a median fold expansion of 71-fold (range, 42-80-fold) at day 14 of culture.

**Table 1:** Donor graft and UCB Treg characteristics

	Patient 1	Patient 2	Patient 3	Patient 4	Patient 5
Day 0 CD25^+^ (10^6^)	25	8.3	12	22	15.5
Day 0 CD25^+^ Purity (%)	70	90	96	83	96
Day 14 TNC (10^6^)	1492	352	857	1267	1242
Day 14 Fold Expansion	60	42	71	58	80
Day 14 % Treg	86	92	93	88	90
Day 14 Pre-Fucosylation CLA (%)	N/A	N/A	3.35	3.5	4.46
Day 14 Post-Fucosylation CLA (%)	N/A	N/A	84.8	79.6	74.7
CB Treg cell dose (1×10^6^ cells/kg)	1.2	1.19	1.1	1.2	1.1
UCB Treg HLA Matching to Recipient	4 out of 6	4 out of 6	5 out of 6	5 out of 6	5 out of 6
Donor Graft HLA Matching to Recipient	CB 1: 4 out of 6 CB 2: 4 out of 6	CB 1: 5 out of 6 CB 2: 5 out of 6	6 out of 6	6 out of 6	6 out of 6
Donor total infused cell dose (1×10^6^ cells/kg)	64.29 (CB#1: 41.27 CB#2: 23.02)	36.95 (CB#1: 21.88 CB#2: 15.07)	1486.25	635.21	2008.61
Donor T cell dose (CD3^+^) (1×10^6^ cells/kg)	28.69 (CB#1: 19.04 CB#2: 9.65)	14.77 (CB#1: 6.74 CB#2: 8.03)	391.70	158.41	371.19
Ratio of Donor Tcon: UCB Treg	24	12	356	132	337

**Figure 1:**
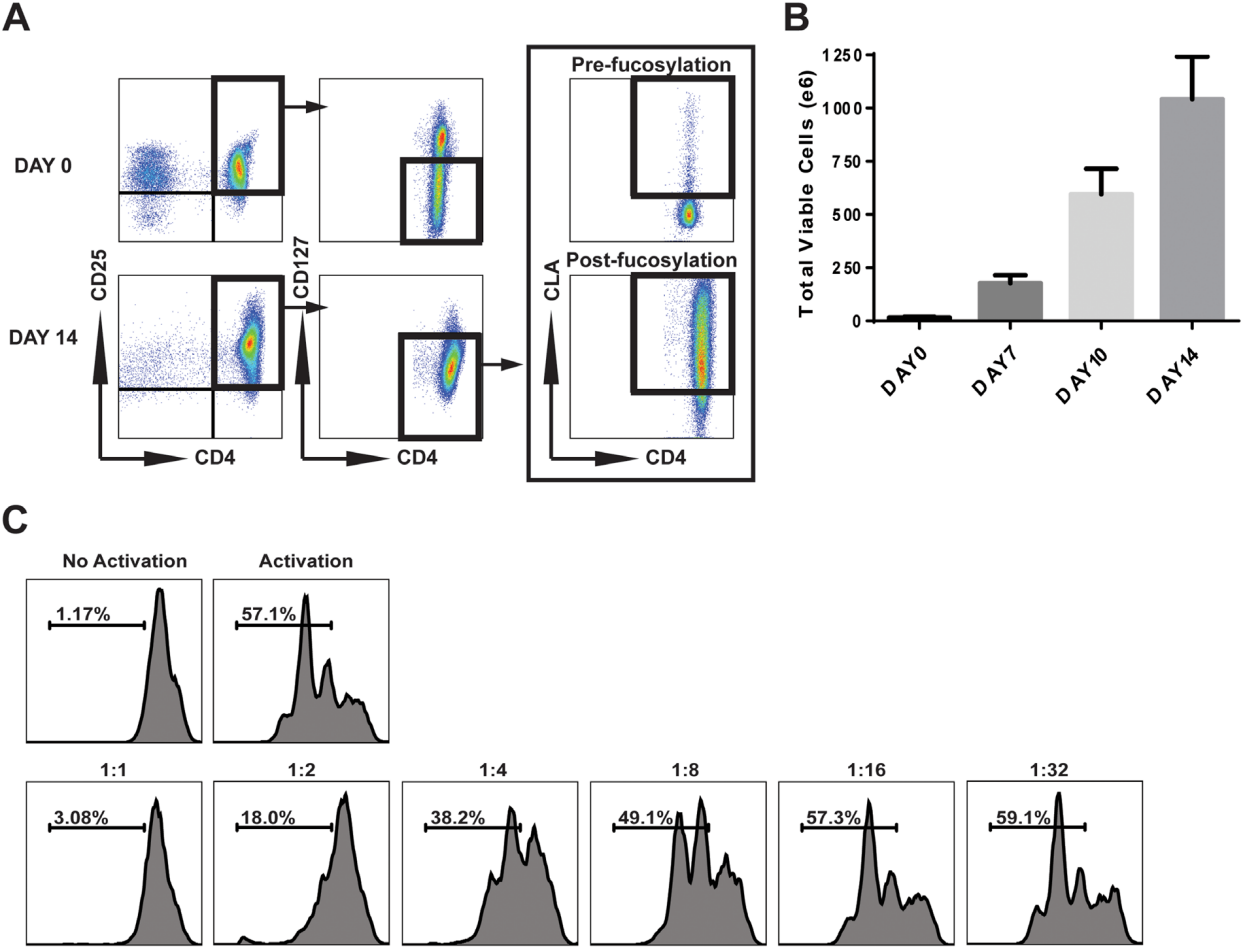
Characterization of clinical *ex-vivo* expanded CB Tregs **(A)** Representative flow cytometry analysis of CB Tregs. Top row is Day 0 isolation of CD25 cells. Bottom row is Day 14 expanded Tregs. Far right panels: CLA expression at Day 14 Pre- (top) and Post- (bottom) fucosylation. **(B)** Total expanded viable cells counted at each time point in culture. Results are mean ± SEM. **(C)** Representative flow plots of Treg:Tcon suppression assay from *ex vivo* expanded CB Tregs.

Figure 1A is a representative of UCB Treg phenotype where far right panels demonstrates CLA expression at Day 14. Figure 1C demonstrates suppressive activity of representative UCB Treg clinical product harvested at Day 14 where the cells maintained significant (>85%) suppressor function. In all patients the ratio of donor T-cells was at least 12 times higher than infused UCB Tregs. Patients did not experience any infusion reaction due to UCB Tregs.

### Patient characteristics (Table 2)

#### Patient 1

**Figure 2:**
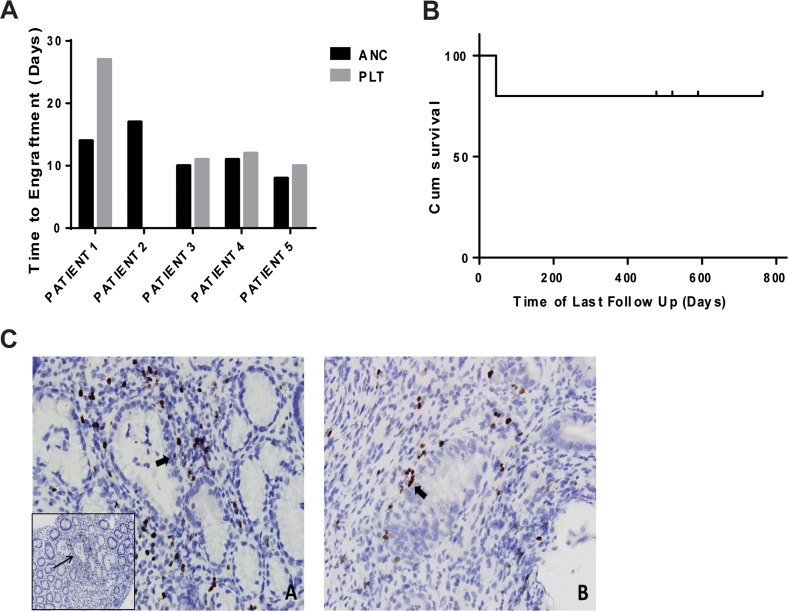
Patient outcomes **(A)** Cumulative Incidence: ANC (Absolute Neutrophil Count) and Platelet engraftment. **(B)** Overall Survival. **(C)** FOXP3 immunohistochemistry showing Treg infiltration in tissue samples with graft-versus-host disease (GVHD). (A) Treg cells in a duodenal biopsy showing clustering around areas of epithelial distortion by GVHD (inset; thin arrow). Higher power shows exocytosis of Tregs into glandular epithelium (thick arrow). (B) Similar findings were seen in a colonic biopsy. [A: 40x (inset 20x); B: 40x].

59 yo female with FLT3 positive AML, status post induction therapy with cladribine/idarubicin/Ara-C/Sorafenib, underwent dUCBT with conditioning regimen: Flu/Cy/TBI +UCB Tregs at dose: 1×10^6^ cells/kg on day −1 and donor cells on day 0; where ratio of Treg:Tcon was 1:24. Neutrophils engrafted Day 14 and platelets on Day 27 with 100% donor chimerism at day 30. On day +25, grade II acute GVHD in the lower gastrointestinal tract (GI) (only) was recorded. Duodenal biopsy immunohistochemistry (IHC) of FOXP3 revealed Treg clustering around areas of GVHD, with occasional exocytosis into epithelial structures (Figure 2C-A). Similar Treg infiltration was also seen in the colonic biopsy (Figure 2C-B). Although initial episode of GVHD resolved with steroids by day 69, GVHD relapsed at day 322 requiring systemic steroids and oral Ruxolitinib. At last follow up, 32 months months from transplant, patient was alive, disease free, and off immune-suppression.

**Table 2:** Patient characteristics and outcomes

	Patient 1	Patient 2	Patient 3	Patient 4	Patient 5
Age (yrs)	59	58	51	65	34
Gender (M/F)	F	M	M	M	M
Diagnosis	Acute Myeloid Leukemia	Mycosis Fungoides	Multiple Myeloma	Myeloid sarcoma	Acute Myeloid Leukemia
Conditioning regimen^*^	Flu/Cy/TBI	Flu/Cy/TBI	Flu/Mel140	Flu/Mel140	Flu/Mel140
Transplant Type	Double cord	Double cord	PB MUD	PB MUD	PB MUD
Infusion reaction (Y/N)	N	N	N	N	N
Time to Engraftment Neutrophil (Days)^**^	14	17	10	11	8
Time to Engraftment Platelets (Days)^**^	27	N/A	11	12	10
D30 donor Chimerism (%)	100	62	100	100	100
Acute GVHD (Y/N)	Y	N	Y	Y	Y
aGVHD grade	II	0	II	IV	II
aGVHD site	GI	N/A	Liver	Skin	Skin
Time to aGVHD (Days)^**^	25	N/A	19	40	19
aGVHD resolution at Day 180	Y	N/A	Y	Y	Y
Time to aGVHD resolution (Days)^**^	69	N/A	40	130	124
Alive (Y/N)	Y	N	Y	Y	Y
Last Follow up (Days)^**^	960 (32 months)	45 (1.5 months)	780 (26 months)	720 (24 months)	660 (22 months)
Relapse GVHD	Y	N/A	N	N	N
Time to GVHD Relapse	322	N/A	N/A	N/A	N/A
Disease Relapse	N	N	N	N	N
Disease Response	CR	N/A	CR	CR	CR
Chronic GVHD	N	N/A	N	N	N

^*^Flu/Cy/TBI: Fludarabine 40 mg/M2 IV (D-8 to D-5), cyclophosphamide 50 mg/kg plus Mesna D-6, Total body radiation 2Gy D-4; Flu/Mel140: Fludarabine 40 mg/M2 IV (D-5 to D-2); Melphalan 140 mg/M2 IV D-2.

^**^All days calculated from the day of transplant.

#### Patient 2

58 yo male with mycosis fungoides/Sezary syndrome, multiple lines of treatment including methotrexate, soriatane, intramuscular steroids, adalimumab and extracorporeal photopheresis with subsequent development of leptomeningeal disease and right temporal lobe lymphoma, status post 7 cycles of high dose methotrexate and one cycle of high dose arabinoside with resolution of brain disease. Due to persistent active nodal disease, patient underwent one cycle of CEOP (cyclophosphamide, etoposide, prednisolone and vincristine) and received dUCBT using conditioning regimen: Flu/Cy/TBI + UCB Tregs at dose: 1×10^6^ cells/kg on day −1 and donor cells on day 0; where the ratio of Treg:Tcon was 1:12. Patient engrafted neutrophils on day 17 and had 62% chimerism on day 30. Patient did not develop GVHD but died of brain hemorrhage on day 45.

#### Patient 3

51 yo male with relapsed/refractory kappa light chain multiple myeloma status post at least 7 lines of treatment including two autologous stem cell transplant, underwent PB MUD AlloSCT using conditioning regimen: Flu/Mel + fucosylated UCB Tregs at dose: 1×10^6^ cells/kg on day −1 and donor cells on day 0; where the ratio of Treg:Tcon was 1:356. Patient developed high fevers and rash on day 5 that resolved with high dose steroids (1 mg/kg). Neutrophils engrafted on Day 10 and platelets on Day 11 with 100% donor chimerism at day 30. On day +19, grade II acute GVHD of liver (only) was reported based on biopsy result which resolved with systemic steroids by day 40 and did not require second line therapy. Patient did not have recurrence of GVHD. At last follow up, 26 months from transplant, patient was alive, free of myeloma, without GVHD and off immunesuppressors. No evidence of chronic GVHD.

#### Patient 4

65 yo male with FLT3 positive myeloid sarcoma status post 3 cycles of FLAG-IDA underwent PB MUD AlloSCT using conditioning regimen: Flu/Mel + 3^rd^ party fucosylated UCB Tregs at dose: 1×10^6^ cells/kg on day −1 and donor cells on day 0; where the ratio of Treg:Tcon was 1:132. Patient developed high fever and rash on day 6 that resolved with high dose steroids (1 mg/kg). Patient engrafted neutrophils on day 11 and platelets on day 12 with 100% donor chimerism at day 30. On day +40, grade IV acute GVHD of skin (only) was reported on biopsy result which required treatment with systemic steroids, extra corporeal photopheresis and Ruxolitinib and resolved by day 130. At last follow up, 24 months from transplant, patient was alive, disease free, without GVHD and off immune-suppression. No evidence of chronic GVHD.

#### Patient 5

34 yo male with AML with high risk cytogenetics status post Ida/Ara-C induction and 3 cycles of high dose Ara-C, underwent PB MUD AlloSCT with conditioning: Flu/Mel +fucosylated UCB Tregs at a dose: 1×10^6^ cells/kg on day −1 and donor cells on day 0; where the ratio of Treg:Tcon was 1:337. Patient developed high fever and rash on day 5 that resolved with high dose steroids (1 mg/kg). Patient engrafted neutrophils on day 8 and platelets on day 10 with 100% donor chimerism at day 30. On day +19, grade II acute GVHD of skin (only) was reported based on biopsy result which resolved with systemic steroids. Patient was intolerant of steroids and was switched to oral Ruxolitinib, resolving GVHD by day 124. At last follow up, 22 months from transplant, patient was alive, disease free, without GVHD and off immune-suppression. No evidence of chronic GVHD.

### Survival data

At a median follow up of 25 months (range 1.5-32 months) there were no disease relapse and overall survival was 80% (Figure 2B).

### Correlative assay

#### Flow cytometry

**Figure 3:**
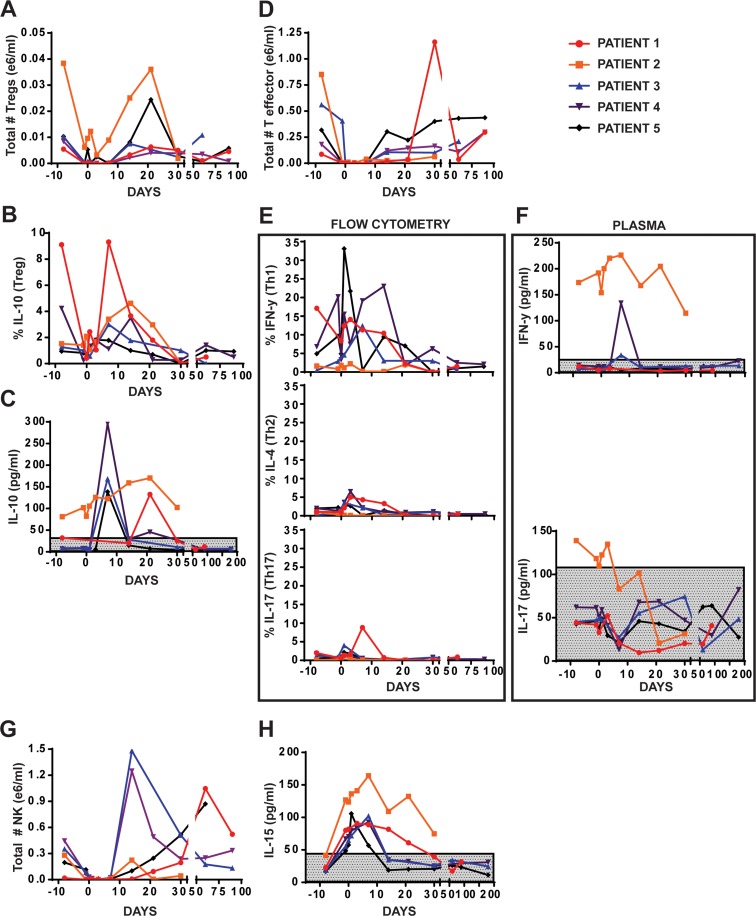
Hematopoietic analysis of patient peripheral blood **(A)** Flow cytometric analysis of number of Tregs (CD4+CD25+CD127-). **(B)** Flow cytometric analysis of percent IL-10 secreting cells in PB. **(C)** Plasma analysis of IL-10 in PB. **(D)** Flow cytometric analysis of T effector cells (RA-RO+CD62L-CCR7-) in PB. **(E-F)** Analysis of Th1 (IFN-γ), Th2 (IL-4), Th17 (IL-17) secreting cell populations using either flow cytometry (E) or Luminex (F). **(G)** Flow cytometric analysis of NK cells in patient PB (CD3-CD56+). **(H)** Plasma analysis of IL-15 in patient PB.

Circulating Tregs showed a concurrent bimodal increase (Day 1, 21) (Figure 3A). IL-10 secreting Tregs were detected at early time points (Day 0,1) with significant increases at Day 7 before subsiding (Figure 3B) while bimodal increase in IL-10 plasma levels occurred at Day 7 and 21 (Figure 3C). No significant changes were observed in naïve T cells and either central or effector memory T-cells, however T-effector (Teff) cell fraction (CD45RA^−^CD45RO^+^CCR7^−^CD62L^−^) increased above normal starting at Day 7 (Figure 3D), resembling a Th1 IFN-γ secreting population with minimal Th2 (IL4) and Th17 (IL-17) populations at early time points (Figure 3E), as evident by higher plasma levels of IFN-γ when compared to IL-17(Figure 3F). An increase in NK cells, CD3^−^CD56^+^CD16^+^, was also observed beginning at Day 7 (Figure 3G) that correlated with elevated IL-15 plasma levels detected in early post-transplantation period (Figure 3H). No statistically relevant increases in dendritic cells or lineage B cells were noted. Extensive immune reconstitution panel is shown in Supplementary Figure 1.

#### Cytokine assay

**Figure 4:**
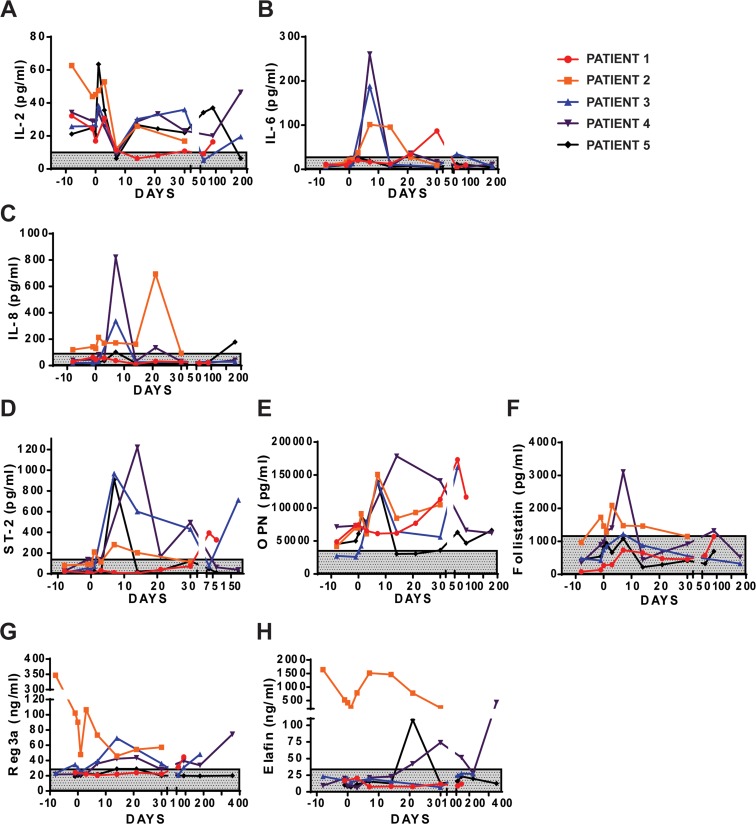
Analysis of GVHD in patient plasma Plasma analysis of inflammatory cytokines IL-2. **(A)** IL-6 **(B)** and IL-8 **(C)** in patient PB. Analysis of GVHD biomarkers ST-2 **(D)**, osteopontin **(E)** and follistatin **(F)** in patient PB. **(G-H)** ELISA analysis of REG3a and Elafin in plasma of patient PB.

Notably, plasma IL-2 dropped in all patients at Day 7 (Figure 4A), whereas, IL-6 and IL-8, both pro-inflammatory cytokines, peaked at Day 7 before decreasing and falling to normal levels at Day 14 (Figure 4B, 4C). While various biomarkers of GVHD were screened, only ST-2, OPN and Follistatin showed elevated levels beginning at Day 7 (Figure 4D, 4E, 4F). Extensive analysis of patient serum is shown in Supplementary Figure 2 (chemokines and growth factors) and Supplementary Figure 3 (cytokines and soluble factors and receptors).

#### Reg3a and elafin assay

Two factors, Reg3a and Elafin, have recently been used as biomarkers of GVHD suggesting involvement in chronic GVHD [3]. Only in PB MUD AlloSCT recipients, the Reg3a slowly increased in the post-transplant period, peaking at Day 14 before subsiding to normal levels (Figure 4G), whereas, Elafin increased later at Days 21-30, before decreasing (Figure 4H). The late increase in the Reg3a and Elafin level in Patient no. 4 was not associated with GVHD relapse or infection. Similarly, no rebound increase was seen in Patient no. 1 who did suffer GI GVHD relapse.

## DISCUSSION

We are the first to show safety and feasibility of infusion of 3^rd^ party, *ex vivo* expanded, fucosylated UCB Treg cells in patients undergoing PB MUD AlloSCT. We had to conduct the study with a low dose of UCB Tregs at 1×10^6^ cells/kg when safety with higher dose has been published by Brunstein et al. [4, 5] due to the recommendation of MDACC safety board, since this was the first time UCB Treg cell product was manufactured at the MDACC GMP facility and the first time UCB underwent fucosylation for clinical use. We understand that with a small sample size with heterogenous characteristics, it is hard to make any concrete dervations, but we can certainly conclude that the UCB Treg infusions were safe without any detrimental effect on the patients. Similarly the different diagnoses and the variable graft characteristics may impact the clinical course and immune reconstitution differently and may prohibit from a conclusive finding. The high variability in the donor T cell: UCB Tregs of 12-356 remained a function of the donor graft characteristics, specifically the low count derived from double cord transplant as compared to the high count reflected in the peripheral blood transplant. Overall, the dose level: 1.0 × 10^6^ cells/kg was well-tolerated with no infusional toxicity or impact on engraftment. Specific presentation of high fevers associated with non-specific inflammatory rash and elevated IL-6 levels in the post-transplant period of patients receiving fucosylated UCB Tregs may be consistent with pre-engraftment syndrome [6, 7]. It is unclear whether the short course of systemic steroids impacted efficacy of infused UCB Tregs, since all patients developed GVHD, however, it is important to consider that the infused donor T cells were significantly higher (12-356 times) than the infused Tregs. Since published clinical data has shown that a higher ratio of Tregs to Tcons is needed for effective prevention of GVHD, we did not expect complete abrogation of GVHD with such a low dose of Tregs. Brunstein et al [4, 5] showed that at least 10 times higher UCB Tregs than Tcons results in decreasing the grade II-IV aGVHD rate to 9% as compared to 45% in controls. Similarly, at least 2 times higher donor Tregs than donor Tcons are needed in the absence of post-transplant immune-suppression in a haplo-transplant setting to result in acute GVHD rate of 15% [8, 9] as compared to 24% in those receiving T-cell replete haplo-transplant [10].

As compared to published data where only a third of patients with grade ≥2 GVHD show durable response to first line therapy [11], in our small sample size, all patients underwent resolution of their GVHD symptoms and were off immune-suppression at a median follow up of 25 months. It is unclear as to why only patient no. 1 developed relapse of GI GVHD and future studies may help us understand whether there is correlation with her high risk AML vs. dietary habits vs. immune reconstitution. At this time we don't have a clear explanation. It is also unclear as to why patient no. 4 who developed steroid refractory grade 4 skin only GVHD at Day +60 requiring photopheresis and oral Ruxolitnib then underwent complete resolution and at 20 months post-transplant, demonstrates no evidence of GVHD with no need for continued immune suppression and remains free of his high risk leukemia. This is notable considering that patients with steroid refractory grade 4 skin GVHD have been reported to have high treatment related mortality [12] Again, larger cohort of patients will allow for valuable insights into the immune reconstitution and may unravel novel mechanisms of GVHD pathogenesis and resolution.

In fact, four out of five patients with high risk disease are alive at a median follow up of 25 months. A recent meta-analysis of fourteen eligible studies where high levels of Tregs in donor grafts were associated with improved OS, a significant reduction in non-relapse mortality and a reduced risk of acute GVHD [13] and supports our findings. Haplo-transplant recipients with high risk leukemia who received adoptive therapy with donor Tregs without post-transplant immune-suppression also showed low risk of relapse of <5% at 3 year follow up [8, 9].

In our report, all patients showed robust immune reconstitution. Infused UCB Tregs are most likely responsible for initial rise in circulating Tregs followed by a second peak due to proliferation of Tregs in response to effector T-cell expansion. Parallel decrease in plasma IL-2 level correlates with expansion of Treg compartment in early post-transplant period, while increase in IL-6 and IL-8 may represent post-transplant inflammatory environment especially in patients with high fever and rash [14]. While increases in IFN-γ secreting cells (Th1) might have also followed initial expansion of T-cell compartment, conflicting data exists for role of IFN-γ both in promoting and inhibiting GVHD [15].

We noted an increase in some GVHD biomarkers including ST2, OPN and Follistatin [16–20] approximately at Day +7, correlating with subsequent development of GVHD. We utilized the algorithm developed by Hartwell *et al.* where concentrations of two biomarkers (ST2 and REG3a) at 7 days post AlloSCT, predicted patients at high risk for lethal GVHD and non-relapse mortality [3]. In our patients normal values of REGa/Elafin at day 7 correlated with lack of chronic GVHD. With enrollment of additional patients in this trial, we will be able to further ascertain the predictive role of such biomarkers.

Although, our study consisted of only 5 patients, the long term follow up of the 4 alive patients who exhibited no infusion reaction or long term complication from UCB Treg infusion and who remain in disease remission and off immune-suppression underscores the promise of cell therapy such as UCB Tregs in the continued improvement of the outcomes of AlloSCT. The next cohort will enroll 10 patients undergoing AlloSCT and will receive non-fucosylated UCB Tregs at dose level: 1×10^7^cells/kg. In this cohort, we will plan to primarily include the PB MUD transplant graft in order to make an attempt to keep the donor T cell: UCB Treg ratio consistent and continue to perform immune reconstitution at the close data points in the post-transplant recovery period. At the time of submitting this manuscript, we have already treated one patient undergoing PB MUD for CML Blast crises with the higher dose of UCB Treg at 1×10^7^cells/kg and at a follow up of 1.5 years, we have observed no evidence of GVHD or leukemia relapse (data not shown). We believe that 3^rd^ party UCB derived Treg therapy holds the promise to be an effective strategy to improve overall outcomes of AlloSCT and additional studies have to be performed to determine the optimal dose.

## MATERIALS AND METHODS

### Treatment and supportive care

The protocol was approved by the Institutional Review Board of University of Texas MD Anderson Cancer Center and registered at https://www.clinicaltrials.gov as NCT02423915. All patients provided written informed consent. Treatment details are shown in Table 2. GVHD prophylaxis included mycophenolate-mofetil 1.5 gram intravenously (IV) or orally (PO) twice daily from day −3 to +30 in combination with oral sirolimus with loading dose of 12 mg followed by 4 mg daily and target through level between 3-12 ug/mL from day −3 to day +100. Supportive care was followed as per institutional guidelines. Diagnosis of acute GVHD was made within 100 days of transplant and chronic GVHD thereafter [21]. GVHD diagnosis was based on patient presentation of acute diarrhea, rash and/or liver function abnormalities followed by tissue biopsy.

### Study objectives

#### Primary objective

Determine safety of administering fucosylated and non-fucosylated UCB Tregs in AlloSCT setting.

#### Secondary objectives

Estimate probabilities of Grade II-IV GVHD; Engraftment; Relapse incidence and survival.

#### Patient inclusion criteria

i) High risk hematologic malignancies; ii) availability of matched related donor (MRD) or MUD source or UCB unit(s) available for the primary transplant., iii) Age ≥16 and ≤80 years, iv) Bilirubin ≤1.5 mg/dl, SGPT ≤200 IU/ml (unless Gilbert's syndrome), v) Creatinine clearance of >50 mL/min, vi) Diffusing capacity for carbon monoxide ≥45%, vii) Left ventricular ejection fraction ≥40%, vii) Zubrod performance status ≤2 [22]), viii) Twenty-one or more days since patient's last radiation or chemotherapy administration before beginning treatment for AlloSCT.

#### Patient exclusion criteria

i) HIV seropositivity, ii) Uncontrolled infection, iii) Pregnancy.

### UCB Treg manufacture

UCB Tregs were manufactured as described previously [1]. Cryopreserved UCB units matched at HLA 3/6 to recipient were provided by MDACC UCB bank. Units were thawed and processed in CliniMACS buffer (Miltenyi Biotec, Bergish Gladbach, Germany) containing 0.5% HSA (Baxter Healthcare, Westlake Village, CA. USA) and mono-nuclear cells selected for CD25^+^ using magnetic beads according to manufacturer's instructions (Miltenyi Biotec, Bergish Gladbach, Germany) [23]. CD25^+^ cells were co-cultured with CD3/28 co-expressing Dynabeads® (Clin *Ex Vivo* ™ CD3/CD28, Invitrogen, Carlsbad, CA) at 1:1 cell:bead ratio and seeded at 1×10^6^ cells/ml in *EX-VIVO* 15 medium (Cambrex BioScience, Walkersville, MD) supplemented with 10% human AB serum (Gemini Bio-Products, Sacramento, CA), 2 mM L-glutamine (Sigma, St. Louis, MO), 1% Penicillin-Streptomycin (Gibco/Invitrogen, Grand Island, NY)] and 1000 IU/ml interleukin (IL)-2 (CHIRON Corporation, Emeryville, CA). Cells were cultured in tissue culture flasks at 37°C/5% CO_2_ incubator and maintained at 1×10^6^ cells/ml with fresh medium and IL-2 every 48-72 hours. Culture was harvested at 14 days. Release criteria included: Viability ≥70%, Endotoxin <5EU/kg, Gram Stain: No organism seen, Mycoplasma: Negative, Sterility: No organism at time of infusion, CD4^−^CD8^+^ cells: <10 %, CD4^+^CD25^+^ cells >60% and <100 beads per 3×10^6^ cells.

### *Ex-vivo* fucosylation

Harvested UCB Treg cells were incubated in fucosylation solution (1/25 dilution of fucosyltransferase enzyme (FTVI) provided by Targazyme Inc. (Carlsbad, CA) in 1 mM GDP Fucose, phosphate-buffered saline 1% HSA) for 30 minutes at room temperature.

### Flow cytometry

*Ex vivo* expanded UCB Tregs were analyzed for CD45/CD4/CD8/CD25/CD127 and cutaneous lymphocyte antigen (CLA/HECA-452) expression. Patient PB were obtained at baseline, Day −1, 0, 1, 3, 7, 14, 21, 30, 60 and 90. Analysis was performed using the following markers: a) T-cells: CD45RO/CD45RA/CD127/CD62L/CD3/CD4/CD8/CCR7; b) B-cells: CD19/IgM/CD27/CD24/CD38; c) Dendritic cells: CD123 (plasmacytoid DC), CD11c (myeloid DC); d) Natural Killer (NK) cells: CD3-CD56+CD16+ (NK); CD3+CD56+CD16+ (NKT).

To assay for cytokine-expressing T cell subsets, thawed cells were rested overnight then activated for 5 hours with Cell Stimulation Cocktail (ThermoFisher, Waltham, MA. USA) and stained for Th1/Th2/Th17/Treg subsets using IFN-γ/IL4/IL-17/IL-10 antibodies respectively. Antibodies were obtained from either BD Biosciences (San Jose, CA) or eBioscience (ThermoFisher). Cells were acquired on an LSR Fortessa (BD Bioscience) and analyzed using FlowJo software (FlowJo, Ashland, OR. USA).

### Suppression assays

Conventional T cells (Tcon cells, CD4^+^CD25^−^) were stained with CellTrace Violet (Invitrogen, Carlsbad, CA) following manufacturer's recommendations and stimulated with 1:1 CD3/28 beads (Invitrogen, Carlsbad, CA) at 50,000 cells/well of a 96-well plate, in presence of Tregs at a different ratios; and then incubated for three days at 37°C followed by assessment of Tcon-proliferation using LSR Fortessa and analyzed using FlowJo software.

### Cytokine array

Cytokine analyses were performed using multiplex immuno-assay platform (Luminex, Austin, TX). Measurements were performed in undiluted samples, except for EPO-R (1:10) and E-Selectin, Elastase, Granzyme B, CCL28, and SDF1a (1:100). Heterblock (Omega Biologicals, Bozeman, MT) was used to prevent interference from heterophilic antibodies. Data were analyzed by 5-parametric curve fitting using Bio-Plex Manager software, version 6.1 (Bio-Rad, Hercules, CA). Multiplex immunoassays were in-house validated and show an intra- and inter-assay variability of <5% and 20%, respectively, and recovery values of spikes proteins in serum of 95–105%.

### Reg3a and elafin

Elisa kits were obtained for Reg3a and Elafin from R&D Systems (Minneapolis, MN). Elisa was performed following manufacturer's instructions. Plasma samples from patients were diluted 1:1200 prior to evaluation and standard curve was utilized to properly determine levels of factors within the plasma. Elisa was analyzed using an Epoch Microplate Spectrophotometer (Biotek, Winooski, VT).

### Immunohistochemistry

Immunohistochemistry (IHC) was performed on automated Leica Bond immunostainers (Leica Biosystems, Buffalo Gove, IL,) as described previously [6]. Briefly, formalin-fixed paraffin-embedded tissue sections cut at 4-5 μm thickness were de-paraffinized and subjected to antigen retrieval prior to incubation with an anti-FOXP3 monoclonal antibody (BioLegend, San Diego, CA. USA). Stains were visualized with 3,3′-diaminobenzidine and hematoxylin counterstain and interpreted by a board-certified pathologist [24]. The intensity of tissue infiltration by Tregs (FOXP3^+^) was expressed as number of positive cells per microscopic 40x high-power field evaluated on an Olympus BX41 light microscope (Leica Biosystems, Wetzlar, Germany).

## SUPPLEMENTARY MATERIALS FIGURES



## References

[1] Parmar S, Liu X, Tung SS, Robinson SN, Rodriguez G, Cooper LJ, Yang H, Shah N, Yang H, Konopleva M, Molldrem JJ, Garcia-Manero G, Najjar A (2014). Third-party umbilical cord blood-derived regulatory T cells prevent xenogenic graft-versus-host disease. Cytotherapy.

[2] Parmar S, Liu X, Najjar A, Shah N, Yang H, Yvon E, Rezvani K, McNiece I, Zweidler-McKay P, Miller L, Wolpe S, Blazar BR, Shpall EJ (2015). Ex vivo fucosylation of third-party human regulatory T cells enhances anti-graft-versus-host disease potency in vivo. Blood.

[3] Hartwell MJ, Özbek U, Holler E, Renteria AS, Major-Monfried H, Reddy P, Aziz M, Hogan WJ, Ayuk F, Efebera YA, Hexner EO, Bunworasate U, Qayed M (2017). An early-biomarker algorithm predicts lethal graft-versus-host disease and survival. JCI Insight.

[4] Brunstein CG, Miller JS, Cao Q, McKenna DH, Hippen KL, Curtsinger J, Defor T, Levine BL, June CH, Rubinstein P, McGlave PB, Blazar BR, Wagner JE (2011). Infusion of ex vivo expanded T regulatory cells in adults transplanted with umbilical cord blood: safety profile and detection kinetics. Blood.

[5] Brunstein CG, Miller JS, McKenna DH, Hippen KL, DeFor TE, Sumstad D, Curtsinger J, Verneris MR, MacMillan ML, Levine BL, Riley JL, June CH, Le C (2016). Umbilical cord blood-derived T regulatory cells to prevent GVHD: kinetics, toxicity profile, and clinical effect. Blood.

[6] Park M, Lee SH, Lee YH, Yoo KH, Sung KW, Koo HH, Kang HJ, Park KD, Shin HY, Ahn HS, Chung NG, Cho B, Kim HK, Korean Cord Blood Transplantation Working Party (2013). Pre-engraftment syndrome after unrelated cord blood transplantation: a predictor of engraftment and acute graft-versus-host disease. Biol Blood Marrow Transplant.

[7] Lee YH, Rah WJ (2016). Pre-engraftment syndrome: clinical significance and pathophysiology. Blood Res.

[8] Di Ianni M, Falzetti F, Carotti A, Terenzi A, Castellino F, Bonifacio E, Del Papa B, Zei T, Ostini RI, Cecchini D, Aloisi T, Perruccio K, Ruggeri L (2011). Tregs prevent GVHD and promote immune reconstitution in HLA-haploidentical transplantation. Blood.

[9] Martelli MF, Di Ianni M, Ruggeri L, Falzetti F, Carotti A, Terenzi A, Pierini A, Massei MS, Amico L, Urbani E, Del Papa B, Zei T, Iacucci Ostini R (2014). HLA-haploidentical transplantation with regulatory and conventional T-cell adoptive immunotherapy prevents acute leukemia relapse. Blood.

[10] Guan L, Li X, Wei H, Gu Z, Zhao S, Zhu C, Yang N, Wang F, Luo L, Gao Z, Huang W, Li H, Wang Q (2018). T Cell-Replete Haploidentical Peripheral Blood Hematopoietic Cell Transplantation for Treatment of T-Lymphoblastic Lymphoma. Ann Transplant.

[11] MacMillan ML, DeFor TE, Weisdorf DJ (2012). What predicts high risk acute graft-versus-host disease (GVHD) at onset?: identification of those at highest risk by a novel acute GVHD risk score. Br J Haematol.

[12] Alam N, Atenafu EG, Tse G, Viswabandya A, Gupta V, Kim D, Lipton JH, Messner HA, Kuruvilla J (2013). Limited benefit of pentostatin salvage therapy for steroid-refractory grade III-IV acute graft-versus-host disease. Clin Transplant.

[13] Fisher SA, Lamikanra A, Dorée C, Gration B, Tsang P, Danby RD, Roberts DJ (2017). Increased regulatory T cell graft content is associated with improved outcome in haematopoietic stem cell transplantation: a systematic review. Br J Haematol.

[14] Ferrà C, de Sanjosé S, Gallardo D, Berlanga JJ, Rueda F, Marìn D, de la Banda E, Ancìn I, Peris J, Garcìa J, Grañena A (1998). IL-6 and IL-8 levels in plasma during hematopoietic progenitor transplantation. Haematologica.

[15] Wang H, Asavaroengchai W, Yeap BY, Wang MG, Wang S, Sykes M, Yang YG (2009). Paradoxical effects of IFN-gamma in graft-versus-host disease reflect promotion of lymphohematopoietic graft-versus-host reactions and inhibition of epithelial tissue injury. Blood.

[16] Betts B, Anasetti C, Pidala J (2015). Biomarkers for GVHD prognosis. Lancet Haematol.

[17] Chen YB, Cutler CS (2013). Biomarkers for acute GVHD: can we predict the unpredictable?. Bone Marrow Transplant.

[18] Ito S, Barrett AJ (2015). ST2: the biomarker at the heart of GVHD severity. Blood.

[19] Turcotte LM, DeFor TE, Newell LF, Cutler CS, Verneris MR, Wu J, Howard A, MacMillan ML, Antin JH, Vercellotti GM, Slungaard A, Blazar BR, Weisdorf DJ (2018). Donor and recipient plasma follistatin levels are associated with acute GvHD in Blood and Marrow Transplant Clinical Trials Network 0402. Bone Marrow Transplant.

[20] Zhao F, Zhang Y, Wang H, Jin M, He S, Shi Y, Guo Y, Zhang Y (2011). Blockade of osteopontin reduces alloreactive CD8+ T cell-mediated graft-versus-host disease. Blood.

[21] Warren EH, Deeg HJ (2013). Dissecting graft-versus-leukemia from graft-versus-host-disease using novel strategies. Tissue Antigens.

[22] Oken MM, Creech RH, Tormey DC, Horton J, Davis TE, McFadden ET, Carbone PP (1982). Toxicity and response criteria of the Eastern Cooperative Oncology Group. Am J Clin Oncol.

[23] Tang Q, Bluestone JA (2013). Regulatory T-cell therapy in transplantation: moving to the clinic. Cold Spring Harb Perspect Biol.

[24] Khoury JD, Wang WL, Prieto VG, Medeiros LJ, Kalhor N, Hameed M, Broaddus R, Hamilton SR (2018). Validation of Immunohistochemical Assays for Integral Biomarkers in the NCI-MATCH EAY131 Clinical Trial. Clin Cancer Res.

