# Supplementary material for: Third party, umbilical cord blood derived regulatory T-cells for prevention of graft versus host disease in allogeneic hematopoietic stem cell transplantation: feasibility, safety and immune reconstitution
Source: Oncotarget. 2018 Nov 2;9(86):35611–22. doi: 10.18632/oncotarget.26242 (PMC6235025; doi:10.18632/oncotarget.26242)
Supplement: Supplementary file 1 [file oncotarget-09-35611-s001.pdf]

Third party, umbilical cord blood derived regulatory T-cells for prevention of graft versus host disease in allogeneic hematopoietic stem cell transplantation: feasibility, safety and immune reconstitution

SUPPLEMENTARY MATERIALS

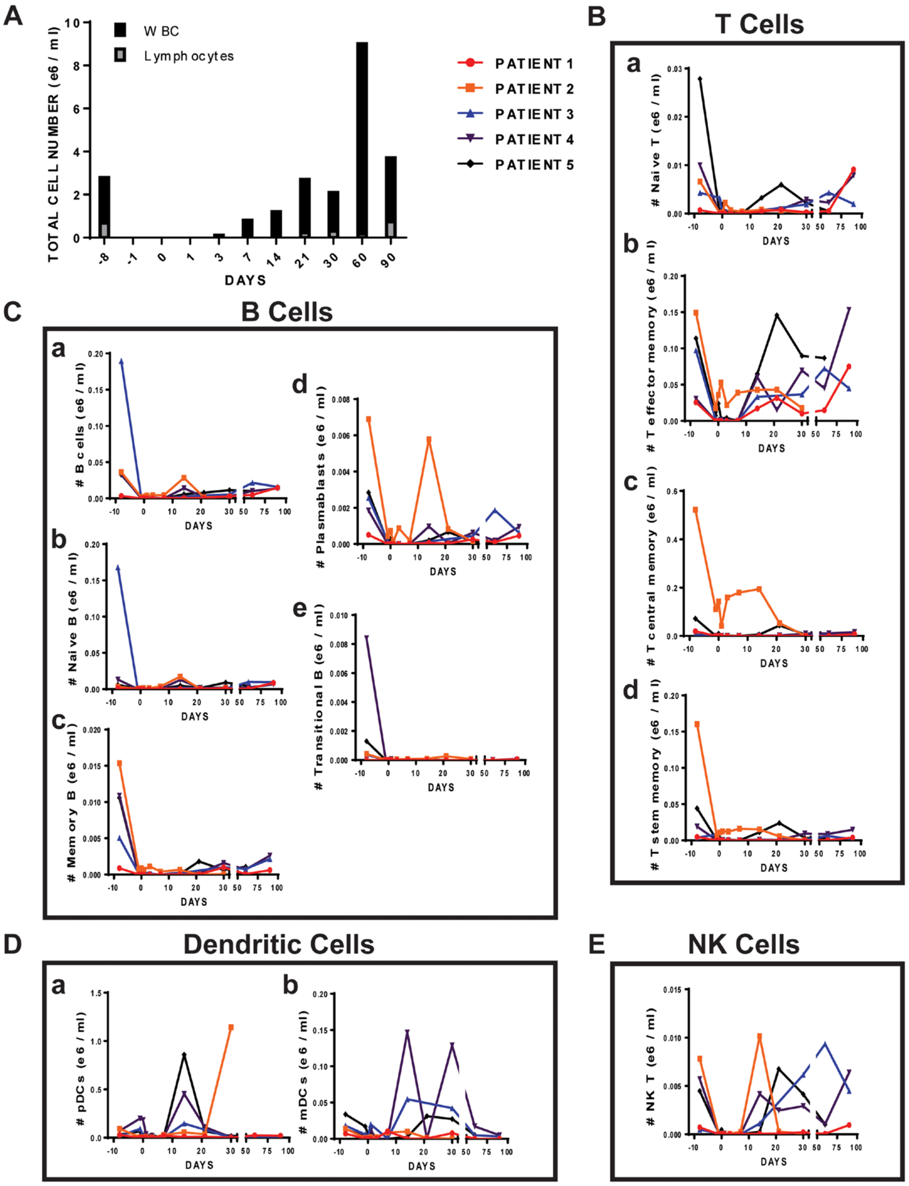

Supplementary Figure 1: Patient cell analysis. (A) Patient white blood cell (black bar) and lymphocyte (gray bar) count. **(B)** T cell analysis. (a) Naïve T cell, RA+RO-CD62L+CCR7-. (b) T effector memory cell, RA-RO+CD62L-CCR7-. (c) T central memory cell, RA-RO+CD62L+CCR7+. (d) T stem memory cell, RA+RO+CD62L+CCR7+. **(C)** B cell analysis. (a) B cells, CD19+. (b) Naïve B cell, CD19+IgM+CD27-. (c) Memory B cell, CD19+IgM-CD27+. (d) Plasmablasts, CD19+CD24-CD38+. (e) Transitional B cells, CD19+CD24hiCD38hi. **(D)** Dendritic cell analysis. (a) plasmacytoid dendritic cells, CD123+. (b) myeloid dendritic cells, CD11c+. **(E)** NK cell analysis. NKT, CD3+CD56+CD16+.

CHEMOKINES

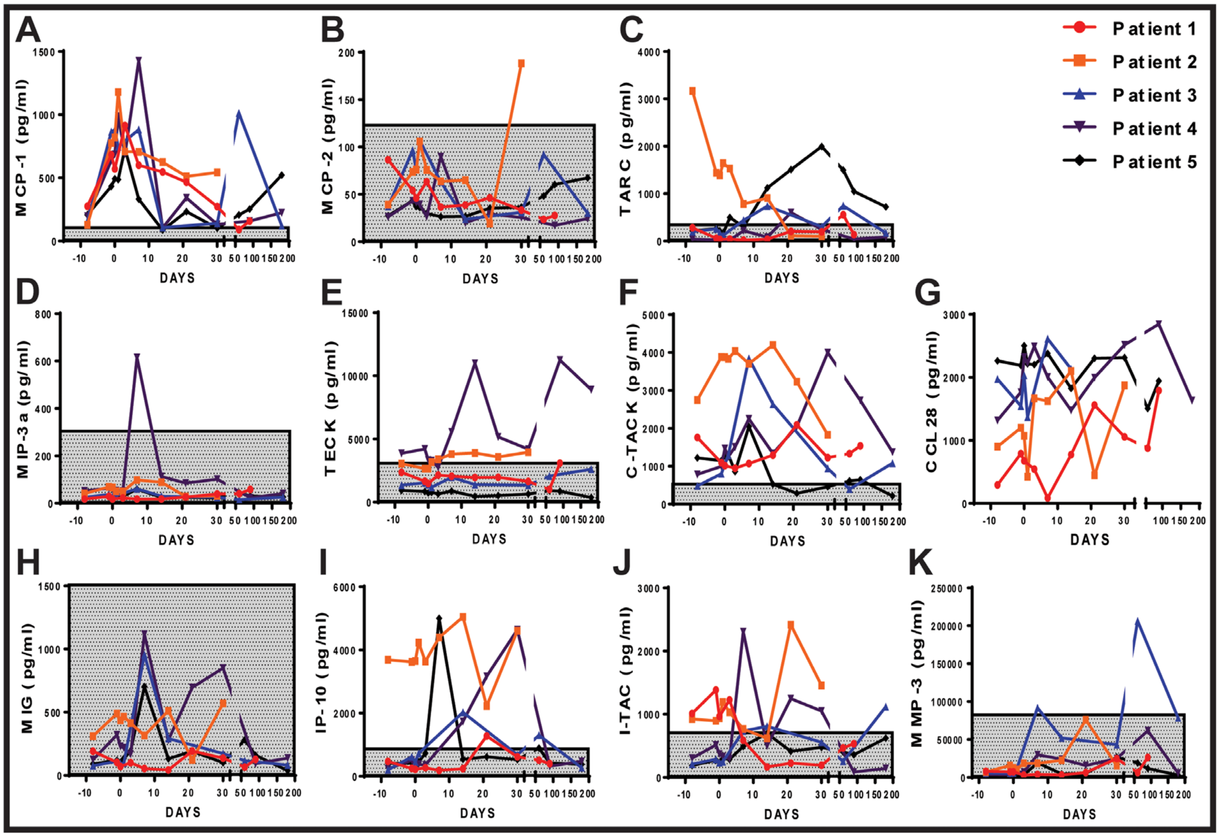

GROWTH FACTORS

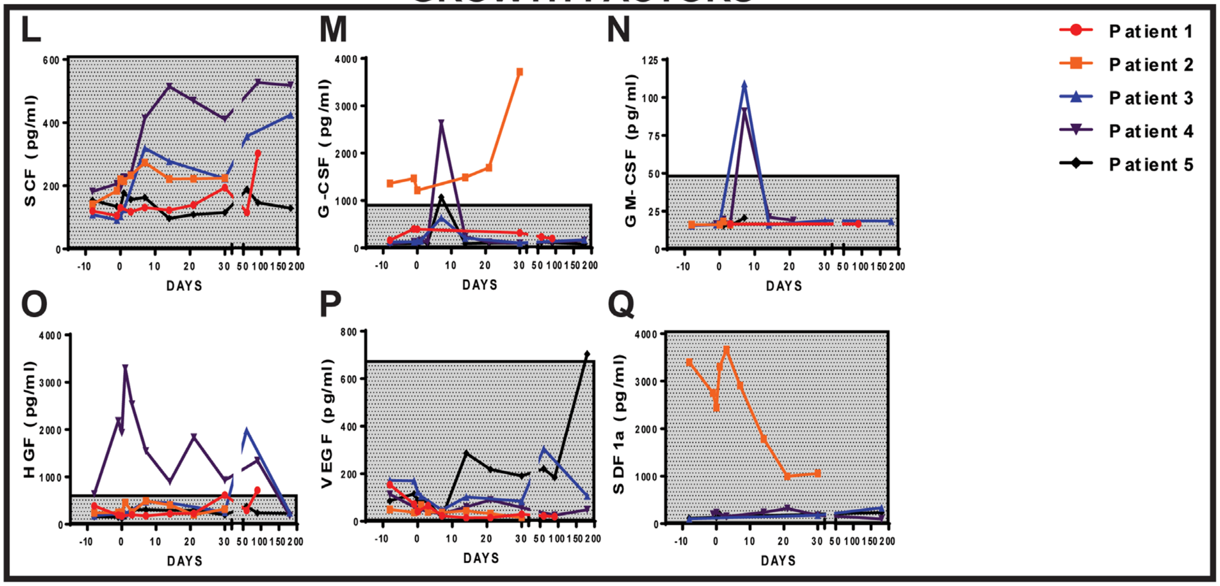

Supplementary Figure 2: Plasma analysis. Chemokines. **(A)** MCP1. **(B)** MCP-2. **(C)** TARC. **(D)** MIP3a. **(E)** TECK. **(F)** CTACK. **(G)** CCL28. **(H)** MIG. **(I)** IP-10. **(J)** I-TAC. **(K)** MMP-3. Growth Factors. **(L)** SCF. **(M)** G-CSF. **(N)** GM-CSF. **(O)** HGF. **(P)** VEGF. **(Q)** SDF1a.

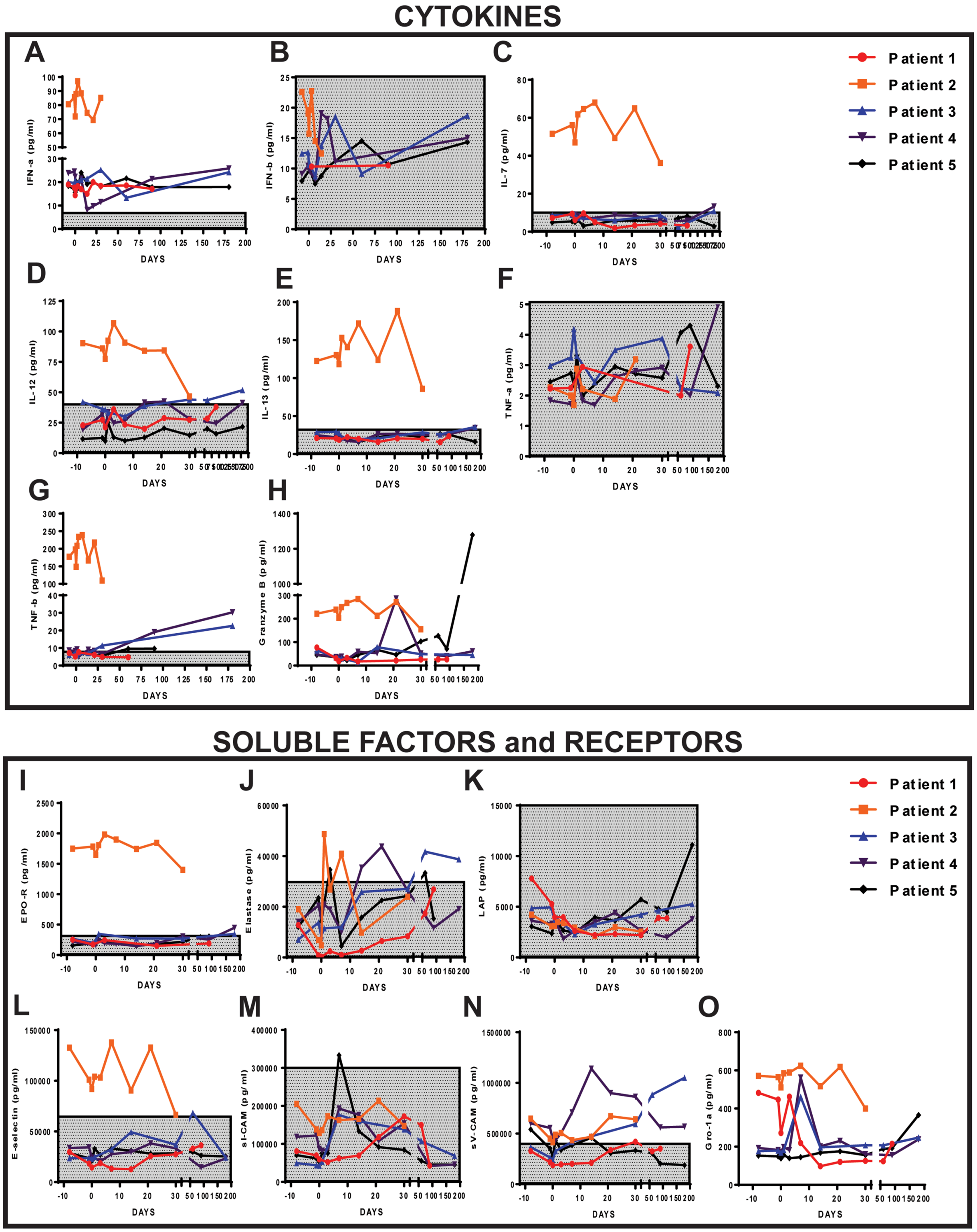

Supplementary Figure 3: Plasma analysis. Cytokines. **(A)** IFN-α. **(B)** IFN-β. **(C)** IL-7. **(D)** IL-12. **(E)** IL-13. **(F)** TNF-a. **(G)** TNF-b. **(H)** Granzyme B. Soluble Factors and Receptors. **(I)** EPO-R. **(J)** Elastase. **(K)** LAP. **(L)** E-selectin. **(M)** soluble I-CAM. **(N)** soluble V-CAM. **(O)** Gro-1a.
